# The Adipokine Visfatin Modulates Cancer Stem Cell Properties in Triple-Negative Breast Cancer

**DOI:** 10.3390/biomedicines11020297

**Published:** 2023-01-20

**Authors:** Yi-Fen Chiang, Ko-Chieh Huang, Hsin-Yuan Chen, Tsui-Chin Huang, Mohamed Ali, Hsin-Yi Chang, Tzong-Ming Shieh, Yin-Hwa Shih, Kai-Lee Wang, Yun-Ju Huang, Cheng-Pei Chung, Shih-Min Hsia

**Affiliations:** 1 School of Nutrition and Health Sciences, College of Nutrition, Taipei Medical University, Taipei 110301, Taiwan; 2 Graduate Institute of Cancer Biology and Drug Discovery, College of Medical Science and Technology, Taipei Medical University, Taipei 110301, Taiwan; 3 Clinical Pharmacy Department, Faculty of Pharmacy, Ain Shams University, Cairo 11566, Egypt; 4 Graduate Institute of Medical Science, National Defense Medical Center, Taipei 114201, Taiwan; 5 School of Dentistry, College of Dentistry, China Medical University, Taichung 40402, Taiwan; 6 Department of Healthcare Administration, Asia University, Taichung 41354, Taiwan; 7 Department of Nursing, Ching Kuo Institute of Management and Health, Keelung 20301, Taiwan; 8 Department of Biotechnology and Food Technology, Southern Taiwan University of Science and Technology, Tainan City 710301, Taiwan; 9 Department of Nutrition and Health Sciences, College of Human Ecology, Chang Gung University of Science and Technology, Taoyuan 333324, Taiwan; 10 Research Center for Food and Cosmetic Safety, College of Human Ecology, Chang Gung University of Science and Technology, Taoyuan 333324, Taiwan; 11 School of Food and Safety, Taipei Medical University, Taipei 110301, Taiwan; 12 Nutrition Research Center, Taipei Medical University Hospital, Taipei 110301, Taiwan; 13 Graduate Institute of Metabolism and Obesity Sciences, College of Nutrition, Taipei Medical University, Taipei 110301, Taiwan; 14 TMU Research Center for Digestive Medicine, Taipei Medical University, Taipei 110301, Taiwan

**Keywords:** visfatin, stemness, triple-negative breast cancer

## Abstract

Obesity is a cancer progression risk factor; excessive adipocytes increase adipokine secretion. Visfatin, a novel adipokine highly expressed in cancer patients, is related to breast cancer risk. The modulation of nicotinamide adenine dinucleotide (NAD+) metabolism and the induction of a tumorigenic environment plays a vital role in cancer progression. Among cancer cell types, cancer stem-like cells (CSCs) with self-renewal and chemotherapy-resistance abilities could modulate tumor progression and cancer recurrence ability. In this study, we focused on visfatin’s modulation effect on stemness-related properties using the high-malignancy breast cancer cell line MDA-MB-231 in in vitro and in vivo studies. Visfatin treatment significantly increased both the sphere number and sphere diameter and increased the protein expression of NANOG homeobox (NANOG), sex-determining region Y-box 2 (SOX2), and octamer-binding transcription factor 4 (OCT4), as well as SIRT1 protein levels. The serum angiogenesis marker VEGF and extracellular nicotinamide phosphoribosyl transferase (NAMPT, visfatin) were induced after visfatin treatment, increasing the stemness and angiogenesis environment, which were significantly reduced by the visfatin inhibitor FK866. Our results demonstrate that the visfatin-activated SIRT–SOX2 axis promotes triple-negative breast cancer stemness and enriches the tumorigenic microenvironment.

## 1. Introduction

Breast cancer is considered the most common cancer worldwide [1]. Among breast cancer types, triple-negative breast cancer lacks estrogen receptor (ER), progesterone receptor (PR), and human epidermal growth factor receptor 2 (HER2) expression [2]. It is characterized by poor prognosis and high drug resistance, resulting in low survival rates. Therefore, it is important to find new therapeutic targets for this cancer.

Obesity is one of the risk factors of breast cancer progression because it leads to an increase in secreted factors [3]. An elevated BMI increases the risk factors for breast cancer, including inflammatory effects and metastasis ability [4]. Obese cancer patients present with larger tumor sizes and higher tumor grades [5]. The elevation of adipokine levels plays an important role in cancer progression [6]. Adipokines induce cancer progression and the epithelial to mesenchymal transition [7] by modulating angiogenesis, invasion, and chemoresistance [8]. The novel adipokine visfatin, also known as nicotinamide phosphoribosyl transferase (NAMPT), shows increased levels in obesity, gynecological diseases, and breast cancer patients [9,10]. The intracellular form of NAMPT is a rate-limited enzyme in NAD^+^ biosynthesis, while the serum visfatin level (extracellular form) is a potential indicator of poor prognosis [11]. An increase in NAD^+^ is related to tumor drug resistance and improvements in DNA repair progression [12]. Among all breast cancer types, MDA-MB-231 showed the highest intracellular and extracellular NAMPT expression [13]. This expression may by elevated by adipose-derived stem cell secretion [14] and tumor-associated macrophages [13], with the supportive environment and intracellular NAMPT expression providing a malignant environment [15].

Since NAD^+^ metabolism is composed of cADP-ribose synthases, ADP-ribosyltransferases and sirtuins such as Sirtuin 1 (SIRT1) are elevated in response to visfatin in cancer stem cells (CSCs) [16]. This results in enhanced self-renewal and cancer metastasis abilities [10] through the octamer-binding transcription factor 4 (OCT4)–SIRT1–p53 axis [11]. Cancer stem-like cells, a subset of tumor cells with self-renewal and chemotherapy resistance abilities, could modulate tumor progression and cancer recurrence ability [17]. The activation of transcription factors is an important regulator of stemness. NANOG homeobox (NANOG), sex determining region Y-box 2 (SOX2), and OCT4, common regulators of stemness, could mediate cancer proliferation and metastasis, which are associated with poor overall survival and advanced disease stage [18]. Our study aimed to determine visfatin’s role in breast cancer stemness progression and the novel therapeutic strategy of visfatin inhibition.

## 2. Materials and Methods

### 2.1. Cell Culture

MDA-MB-231 human breast cancer cells were purchased from the Bioresource Collection and Research Center (BCRC, Hsinchu, Taiwan). DMEM/F12 medium was used for cell culturing (CAISSON, Taichung City, Taiwan) supplemented with 10% fetal bovine serum (FBS; CORNING, Manassas, VA, USA), 1% antibiotic-antimycotic solution (CORNING), sodium bicarbonate (2.438 g/L; BioShop, Burlington, ON, Canada), and 4-(2-hydroxyethyl) piperazine-1-ethanesulfonic acid (HEPES; 5.986 g/L; BioShop) in a humidified incubator (37 °C, 5% CO_2_). Visfatin was obtained from Peprotech (#130-09, Rehovot, Israel), and its inhibitor was obtained from Cayman (Ann Arbor, MI, USA).

### 2.2. MTT Assay

We cultured 3 × 10^3^ cells in 96-well plates. After 24 h of serum-free starvation, they were treated with different dosages of visfatin for 24 and 48 h. After treatment, 5 mg/mL of MTT (3-(4,5-dimethyl thiazol)-2,5-diphenyltetrazolium bromide (Abcam, Cambridge, MA, USA) was diluted to 1 mg/mL with culture medium and kept in a 37 °C CO_2_ incubator for 3 h until a purple crystal formed. We added 100 μL of DMSO to dissolve the crystal. An ELISA reader (Molecular Devices, San Jose, CA, USA) was used to detect the absorbance at the wavelengths of 570 and 630 nm.

### 2.3. Sphere Formation Assay

MDA-MB-231 cells were cultured in 6-well plates (1 × 10^5^) and treated with visfatin for 24 or 48 h. Then, they were harvested and suspended as pellets, followed by counting using trypan blue. Then, 500 cells/well were cultured in 96-well ultra-low attachment plates (Corning, Shanghai, China, 3474) in tumor sphere medium (1 × B27 (Life Technologies, Carlsbad, CA, USA), 20 ng/mL epidermal growth factor (Enzo, Beijing, China), 10 ng/mL basic fibroblast growth factor (Sciencell, Carlsbad, CA, USA), 5 μg/ml insulin (Life Technologies, Carlsbad, CA, USA), and 0.4% bovine serum albumin (Sigma-Aldrich, St. Louis, MO, USA) for 7 days. Microscopy was used to capture the sphere formation, and ImageJ software was used to analyze the diameter [19].

### 2.4. Xenograft Animal Model

Five-week-old female Balb/c nude mice (BioLASCO, Taipei, Taiwan) were housed under a 12 h light/12 h dark cycle in a pathogen-free environment with food and water available ad libitum. After 1 week of adaptation, MDA-MB-231-GFP cells (2 × 10^6^ in 100 μL PBS/mice) were injected subcutaneously into the right flank of the mice. After 1 week, the animals were randomly divided into three groups (n = 5/group) and treated with either visfatin (2 ng/g) or FK866 (4 mg/kg) intraperitoneal injections for 56 days or were untreated controls [9]. We used calipers and calculated the tumor size using the formula 0.5 × length × width^2^. All animal studies were conducted according to protocols approved by the Institutional Animal Care and Use Committee (IACUC) of Taipei Medical University (IACUC Approval No. 2019-0034).

### 2.5. Western Blot Analysis

Tumor samples (about 0.1 g) were homogenized with lysis buffer containing a protease inhibitor (Roche, Basel, Switzerland) and a phosphatase inhibitor (Roche) using TissueLyser II (Qiagen, Chatsworth, CA, USA) and centrifuged at 12,000× *g* for 30 min at 4 °C. A BCA kit (T-Pro Biotechnology, New Taipei City, Taiwan) was used to measure the tissue and serum protein concentration. We used 10–15% SDS-polyacrylamide gel electrophoresis (PAGE) for protein separation and then transferred the samples onto Immobilon-P polyvinylidene fluoride (PVDF) membranes (0.22 µm) for 125 min at 95 V. Then, we used blocking buffer (5% BSA) for 1 h at room temperature and incubated the samples with primary antibodies against NAMPT (Proteintech, Rehovot, Israel), NANOG (Proteintech), OCT4 (Proteintech), SOX2 (Proteintech), SIRT1 (Cell signaling, Boston, MA, USA), VEGF (Santa Cruz Biotechnology, Santa Cruz, CA, USA), and glyceraldehyde-3-phosphate dehydrogenase (GAPDH) (Proteintech, Rehovot, Israel) overnight at 4 °C. After washing 3 times, the samples were incubated with anti-rabbit/mouse IgG coupled with alkaline phosphatase (1: 10,000) for 2 h. We used an ECL chemiluminescent kit to visualize the antibody–antigen interaction and detected the signal with an eBlot Touch Imager (eBlot Photoelectric Technology, Shanghai, China) [20]. The relative intensity was measured using ImageJ software (NIH, Bethesda, MD, USA).

### 2.6. Statistical Analysis

Data are expressed as mean ± standard error of the mean (SEM), and GraphPad Prism version 8.0 (GraphPad, San Diego, CA, USA) was used for the statistical analyses. We utilized Student’s *t*-test, one-way analysis of variance (ANOVA), and Tukey’s post hoc test. *p* < 0.05 was considered statistically significant.

## 3. Results

### 3.1. Effect of Visfatin on Cell Viability

**Figure 1:**
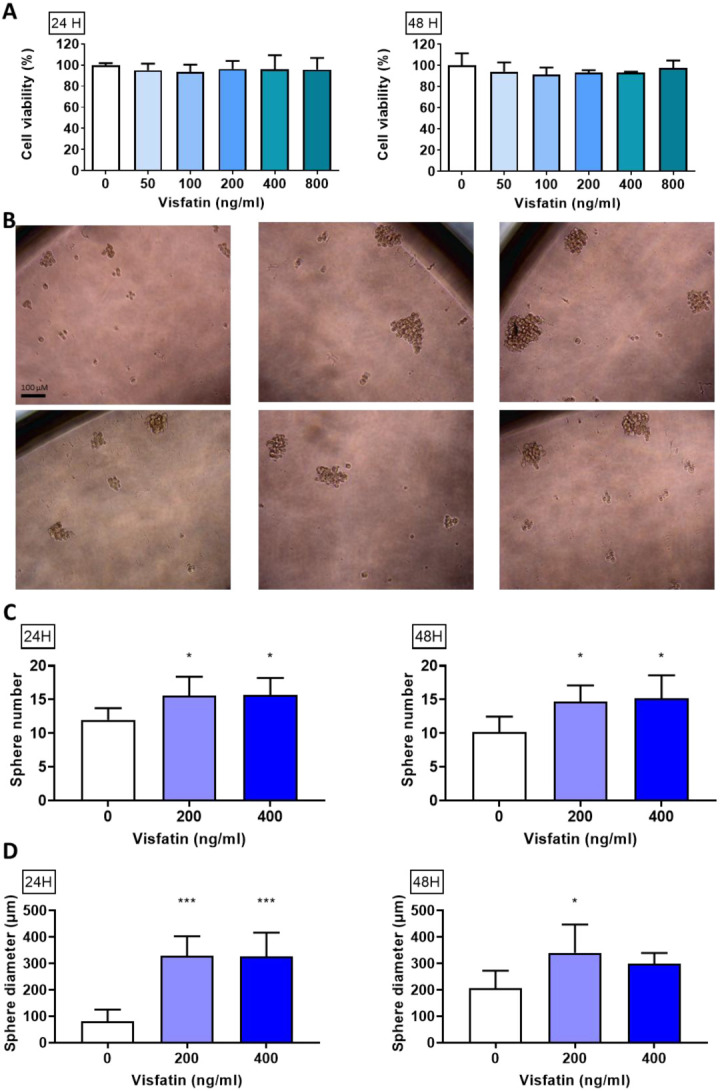
Effect of visfatin on sphere formation in MDA−MB−231. (**A**) Cell viability of MDA−MB−231 after visfatin treatment. (**B**) Sphere morphology after culturing in sphere formation medium for 7 days. (**C**) Sphere number and (**D**) sphere diameter measured using ImageJ. Data are presented as mean ± SD. * *p* < 0.05 and *** *p* < 0.001 as compared with control group.

To explore visfatin’s role in breast cancer cell viability, an MTT assay was used after the 24 or 48 h treatments. There were no statistically significant changes after visfatin treatment, indicating that short-term exposure does not alter proliferation (Figure 1A).

### 3.2. Effect of Visfatin Exposure on Sphere Formation in MDA-MB-231

To evaluate whether visfatin could activate stemness ability and self-renewal in breast cancer, MDA-MB-231 cells were treated with visfatin (0, 200, or 400 ng/mL) for 24 or 48 h (Figure 1B) while using a sphere-forming assay and calculate the sphere number (Figure 1C) and diameter (Figure 1D). Our results showed that visfatin significantly increased the sphere formation ability.

### 3.3. Body Weight and Tumor Size Changes in Visfatin-Exposed Xenograft Animal Model 

**Figure 2:**
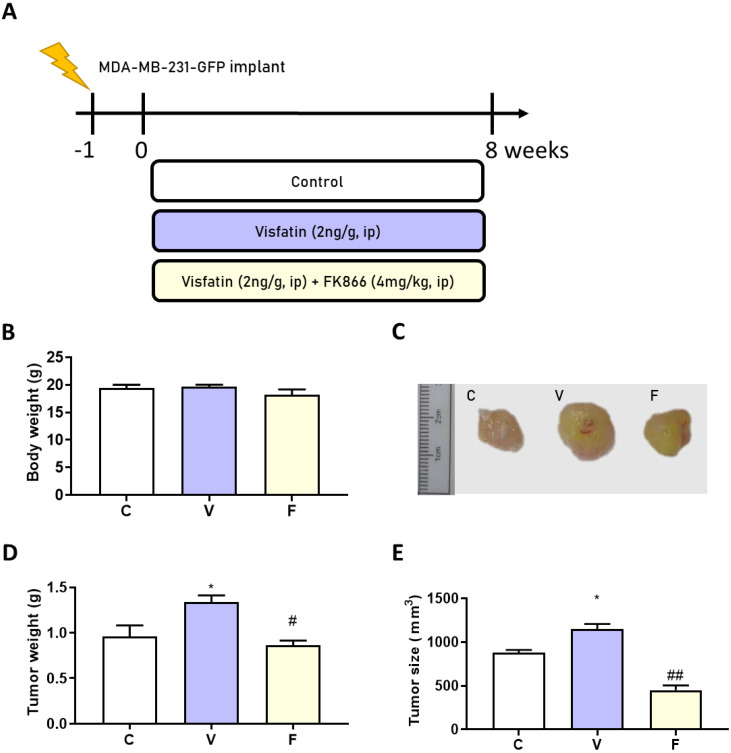
Effects of visfatin and its inhibitor FK866 on an MDA−MB−231−GFP xenograft animal model. (**A**) Flow chart of model induction. (**B**) Change in body weight. (**C**) Tumor morphology. (**D**) Tumor weight change and (**E**) tumor size change after 56 days’ treatment. Data are presented as mean ± SEM. C, control group. V, visfatin−induced group. F, visfatin + FK866 inhibitor group. * *p* < 0.05 compared to C. # *p* < 0.05 and ## *p* < 0.01 compared to V.

To confirm the in vitro results of visfatin-induced tumor-initiating capabilities in vivo, we injected the MDA-MB-231-GFP cell line (2 × 10^6^ cells/100 μL) into Balb/c nude mice. After 1 week, either visfatin alone or visfatin with its inhibitor were given intraperitoneally for 8 weeks (Figure 2A). No significant body weight change was observed between groups (Figure 2B). Interestingly, visfatin treatment significantly increased both tumor size and tumor weight, while treatment with its inhibitor displayed a significant reduction in tumor size and weight (Figure 2C–E). 

### 3.4. Stemness-Related Protein Expression in Visfatin-Exposed Xenograft Animal Model

**Figure 3:**
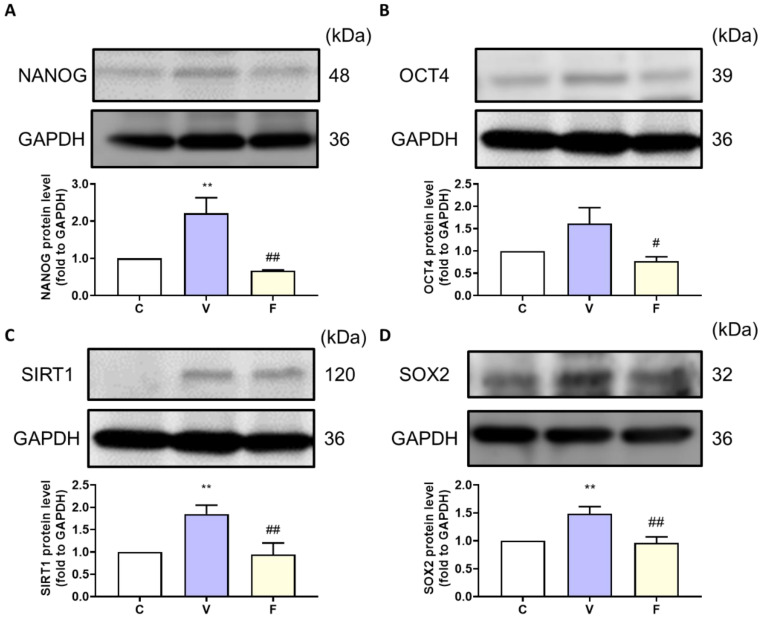
Effect of visfatin on stemness-related protein expression in xenograft tumor tissue. The tumor stemness-related expression of (**A**) NANOG, (**B**) OCT4, (**C**) SIRT1, and (**D**) SOX2 proteins. Data are presented as mean ± SEM. C, control group. V, visfatin-induced group. F, visfatin + FK866 inhibitor group. ** *p* < 0.01 compared to C. # *p* < 0.05 and ## *p* < 0.01 compared to V.

With an increase in tumor weight, we evaluated the stemness markers NANOG, OCT4, and the SIRT1–SOX2 axis in the tumor. Stemness-related protein expression was measured by Western blot analysis. The visfatin-treated group showed induction of stemness-related protein expression, while the visfatin inhibitor FK866 significantly decreased the NANOG, OCT4, SIRT1, and SOX2 protein expression (Figure 3). These results suggest that visfatin, through an increase in NAD^+^, upregulates SIRT1 levels and activates the SIRT–SOX2 axis to modulate stemness progression [21,22].

### 3.5. Extracellular NAMPT and Serum Angiogenesis Marker Changes in Visfatin-Exposed Xenograft Animal Model

**Figure 4:**
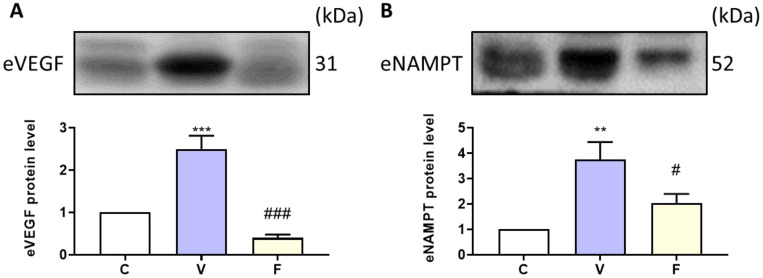
Effect of visfatin on serum angiogenesis marker and extracellular NAMPT expression. The protein expressions of (**A**) VEGF and (**B**) eNAMPT were analyzed by Western blot. Data are presented as mean ± SEM. C, control group. V, visfatin-induced group. F, visfatin + FK866 inhibitor group. ** *p* < 0.01 and *** *p* < 0.001 compared to C. # *p* < 0.05 and ### *p* < 0.001 compared to V.

To explore the effect of visfatin and its inhibitor on extracellular NAMPT and the angiogenesis marker in serum, we used Western blot analysis to measure protein expression. Our results showed that visfatin induction significantly increased serum VEGF and NAMPT levels, while the inhibition of visfatin significantly decreased the cancer-stemness-rich microenvironment (Figure 4).

## 4. Discussion

This work presented the first insights into visfatin’s role in stemness-related markers in a breast cancer animal model with the induction of OCT4, NANOG, and the SIRT1–SOX2 axis. Treatment with the visfatin inhibitor FK866 significantly reversed the induced stemness and decreased the tumor size.

Obesity is one of the known risk factors for breast cancer and is related to worse prognosis and overall survival [23]. Every 5 kg/m^2^ increase in BMI increases breast cancer risk by 2% [24]. In addition, the efficacy of chemotherapy is significantly lower in obese breast cancer patients [25]. The growth of adipocytes increases the secretion levels of cytokines and adipokines, making adipokines one of major contributors to breast cancer progression [26]. Among various adipokines, adiponectin, leptin, resistin, and visfatin are considered to have the greatest relevance to obesity-related cancer [6]. Visfatin, as a relatively newly discovered adipokine, has not been fully explored in the context of tumor progression. Several studies have revealed that the serum visfatin level is highly associated with breast cancer incidence [27]. Additionally, its specificity may qualify it as a diagnostic indicator for breast cancer [28]. In colorectal cancer, visfatin-induced stemness was shown to be related to stem cell signaling transduction and radiotherapy resistance, with a positive correlation being found between stemness-related marker expression and NAMPT expression [29].

Visfatin (NAMPT)’s role in tumorigenesis is attributed to its identity as a rate-limiting enzyme in the salvage pathway [30]. NAMPT’s management involves the recycling of nicotinamide (NAM), where nicotinamide riboside (NR) converts it to nicotinamide mononucleotide (NMN). NMN is enzymatically converted to nicotinamide adenine dinucleotide (NAD^+^). High NAD^+^ levels have been observed in CSCs and could modulate sirtuin function, especially SIRT1 activity, which regulates SOX2-related stemness expression [31]. A reduction in NAD^+^ levels causes self-renewal to decrease and activates apoptosis [32]. Our data suggest that the visfatin-treated tissues showed significantly higher NANOG, OCT4, and SOX2 stemness-related protein expression, with the excessive NAD^+^ production activating SIRT1 expression to enhance SOX2 expression. Moreover, the inhibition of NAMPT through treatment with the visfatin inhibitor FK866 significantly decreased stemness expression and reduced tumor size.

The secreted form of NAMPT (eNAMPT, extracellular NAMPT) has been reported to reflect cytokine function and is associated with cancer and inflammatory disease incidence [33]. Recent studies have revealed that circulating serum eNAMPT was increased in all cancer patients and may be a potential therapeutic target [34]. eNAMPT could increase colony formation ability [35]. However, there is no solid evidence in breast cancer studies to date. Additionally, how eNAMPT is secreted and the modulation between iNAMPT (intracellular NAMPT) and eNAMPT is still not clear. In this study, we measured eNAMPT protein expression and visfatin treatment significantly increased eNAMPT protein expression. FK866 decreased its expression, which is consistent with the pattern of stemness-related protein expression.

Nutrient supply is an important factor to support cancer progression [36]. Therefore, angiogenesis improves tumor growth and migration, as creating a new vascular system enhances the nutrition supply [37]. Adipocyte-derived angiogenic adipokines include VEGF as one of the angiogenesis factors that enriches the tumorigenic microenvironment [36] and increases the number of CSCs [38]. In our study, visfatin treatment increased the serum VEGF level.

Altogether, our data suggest that in breast cancer, the adipokine visfatin increased cancer sphere formation and stemness-related protein expression with SIRT1 modulation and also increased eNAMPT and VEGF levels with subsequent angiogenesis enhancement and enrichment of the tumorigenic microenvironment. Further studies could focus on the signaling pathways that could modulate eNAMPT as a potential novel therapeutic target. The limited sample size makes it difficult to predict the modulatory effect of NAMPT, and further studies should enlarge the sample size to predict the effects of NAMPT and stemness-related genes on tumor size with a mathematical model [39].

## 5. Conclusions

Exposure to the adipokine visfatin may activate the SIRT1-SOX2 axis and stemness progression in breast cancer, as visfatin treatment increased sphere formation and tumor size by activating stemness-related protein expression and increasing angiogenesis, providing a malignant environment for breast cancer progression. The inhibition of visfatin may provide a new therapeutic direction for the treatment of adipokine-related microenvironments in CSCs.
